# An Effective and Fast Model for Characterization of Cardiac Arrhythmia and Congestive Heart Failure

**DOI:** 10.3390/diagnostics15070849

**Published:** 2025-03-27

**Authors:** Salim Lahmiri, Stelios Bekiros

**Affiliations:** 1 Department of Supply Chain and Business Technology Management, John Molson School of Business, Concordia University, Montreal, QC H3H 0A1, Canada; salim.lahmiri@concordia.ca; 2 Valter Cantino Department of Management, University of Turin (UniTo), 10124 Torino, Italy

**Keywords:** electrocardiography, cardiac arrhythmia, congestive heart failure, normal sinus rhythm, discrete cosine transform, classification, CAD system

## Abstract

**Background/Objectives:** Cardiac arrhythmia (ARR) and congestive heart failure (CHF) are heart diseases that can cause dysfunction of other body organs and possibly death. This paper describes a fast and accurate detection system to distinguish between ARR and normal sinus (NS), and between CHF and NS. **Methods:** the proposed automatic detection system uses the higher amplitude coefficients (HAC) of the discrete cosine transform (DCT) of the electrocardiogram (ECG) as discriminant features to distinguish ARR and CHF signals from NS. The approach is tested with three statistical classifiers, of which the k-nearest neighbors (k-NN) algorithm. **Results:** the DCT provides fast compression of the ECG signal, and statistical tests show that the obtained HACs are different from ARR and NS, and for CHF and NS. The latter achieved highest accuracy under ten-fold cross-validation in comparison to Naïve Bayes (NB) and nonlinear support vector machine (SVM). The kNN yielded 97% accuracy, 99% sensitivity, 90% specificity and 0.63 s processing time when classifying ARR against NS, and it yielded 99% accuracy, 99.7% sensitivity, and 99.2% specificity, and 0.27 seconds processing time when classifying HCF against NS. In addition to a fast response, the DCT-kNN system yields higher accuracy in comparison to recent works. **Conclusions:** it is concluded that using the DCT based HACs as biomarkers of ARR and CHF can lead an efficient computer aided diagnosis (CAD) system which is fast, accurate and does not require ECG signal pre-processing and segmentation. The proposed system is promising for applications in clinical milieu.

## 1. Introduction

The electrocardiogram (ECG) is commonly used to evaluate the operation of the cardiac muscle via its electrical signals. In the clinical milieu, the measured electrical activity is examined to detect several heart malfunctions, including cardiac arrhythmia (ARR) and congestive heart failure (CHF). Cardiac arrhythmia refers to the irregular or excessively slow or fast electrical activity of the human heart. This condition can cause serious injury to other organs and may even result in sudden cardiac arrest. Congestive heart failure is a progressive disorder characterized by the heart muscle not pumping enough blood because of thin and weak walls of the ventricles. As a result, several other vital organs can be damaged. Because of their impact on health, the early detection and treatment of ARR and CHF are important for people’s well-being and life expectancy.

Since examining different abnormal variations in lengthy ECG signals is a complex and burdensome task for the clinician, a large number of computer-aided diagnosis (CAD) systems have been proposed in the literature to automatically analyze and classify ECG signals into relevant physiological conditions using a wide range of signal-processing techniques and artificial intelligences tools, especially for distinguishing ARR and normal sinus rhythm and CHF and normal sinus rhythm. A short survey of recent works follows:

For the classification of ARR versus normal sinus rhythm, the authors in [1] propose a modular neural network based on a mixture of experts and negatively correlated learning, trained with ten morphological features and timing intervals reflecting the deviation from a constant beat and achieving 96.02% accuracy. The authors in [2] model the ECG signal by differential equations, with the resulting coefficients employed to train a genetic fuzzy system; 93.34% classification accuracy is reported. In another study [3], statistical features were extracted from spectral correlation coefficients, and principal component analysis and the Fisher score were used for dimension reduction. Using the support vector machine (SVM) classifier with a linear kernel achieved 98.60% accuracy. In [4], the heartbeat is represented by 30 projection coefficients derived from a random projection matrix and the weighted inter-beat (RR) intervals used to train an SVM classifier with a radial basis function kernel. A global accuracy of 98.46% was obtained. More recently, higher-order statistics (HOS) and sample entropy were estimated from selected modes of improved complete ensemble empirical mode decomposition [5]. The AdaBoost classifier yielded 98.6% accuracy.

For the classification of CHR versus normal sinus rhythm, a support vector machine with a linear kernel was trained with static indices and achieved 98.31% accuracy [6], and an ensemble bagged trees classifier achieved 99.5% accuracy, 100% specificity, and 98.6% sensitivity when trained by time-domain heart rate variability statistics and RR features obtained by a probabilistic symbolic pattern recognition model [7]. The authors in [8] assessed the complexity of the RR signal by the part of regularity caused by the non-random aspect of the phase. Based on Student’s t-test, it was found that multi-scale transition of fuzzy sample entropy of RR signals and their phase-randomized surrogates allows individual participants with CHF and healthy controls to be classified with 87% sensitivity and 89% specificity. In yet another interesting work [9], the ECG signal dynamics were examined with the visibility graph method. It was found that the mean power of scale freeness in the visibility graph (PSVG) for CHF patients is 10% lower than for healthy subjects. In addition, an analysis of variance (ANOVA) procedure indicated that the PSVG statistics are significantly different for CHF and the ECG. More recently, the authors in [10] used short-term heartrate variability indices in the time and frequency domains and non-linear statistics in differentiating normal sinus rhythm subjects and CHF patients. The SVM with the radial basis function yielded 90.95% accuracy, 91.31% sensitivity, and 90.04% specificity.

The overview of the recent works [1,2,3,4,5,6,7,8,9,10] on the detection of cardiac arrhythmia and congestive heart failure indicates that high classification accuracy is usually achieved by different approaches, but relatively complex procedures are used, with a potentially negative impact on response time and reliability. Those limitations may be offset by a CAD model of reduced complexity, where the pre-processing and segmentation of the ECG is unnecessary for obtaining the relevant features to describe the heart’s electrical activity. This work proposes such a system. It describes an automatic method that can distinguish between ARR and a normal ECG, as well as between CHF and a normal ECG signal using a fast and analytical signal transform approach. As will be seen, the proposed model is straightforward, effective, and easy to implement.

To differentiate the ARR or CHF signal from a normal ECG, the discrete cosine transform (DCT) [11] is used to decompose the heart signal into harmonic components. The DCT is chosen because of the natural periodic fluctuation of the ECG signal as well as two other desirable features. First, it allows the ECG record to be compressed, eventually shortening the time required for subsequent signal processing. Second, it is fast to execute and effective in highlighting the fluctuations of high amplitude components in the original signal, hence leading to discriminant features with high noise immunity. Finally, the extracted DCT-based high amplitude coefficients are used to train various classifiers.

The rest of the paper is organized as follows. The methods are briefly presented in Section 2. The database and experimental results are presented in Section 3. The experimental results are discussed in Section 4. Finally, we conclude in Section 5.

## 2. Materials and Methods

This section highlights the methodology used in this work. It provides a summary description of the DCT, followed by that of the three different classifiers. In our study, we hypothesize that the first half of the DCT coefficients, which correspond to the higher amplitude components and account for most of the ECG energy, have different values in the presence of ARR and CHR, when compared to those of normal ECG. Indeed, cardiac arrhythmia causes irregular, too slow/fast heart beats and congestive heart failure yields to abnormal blood pressure regulation and variability. Therefore, we expect this variability to be reflected in the high amplitude coefficients. Furthermore, by ignoring the low amplitude upper half of the DCT coefficients with limited information content, the processing time related to the classification task is reduced. 

The vector composed of high amplitude coefficients is used to train the k-nearest neighbors (kNN) algorithm [12]. The latter is a nonparametric supervised classifier that is easy to implement and understand, as the classification is based on the closest training examples in the feature space.

**Figure 1:**
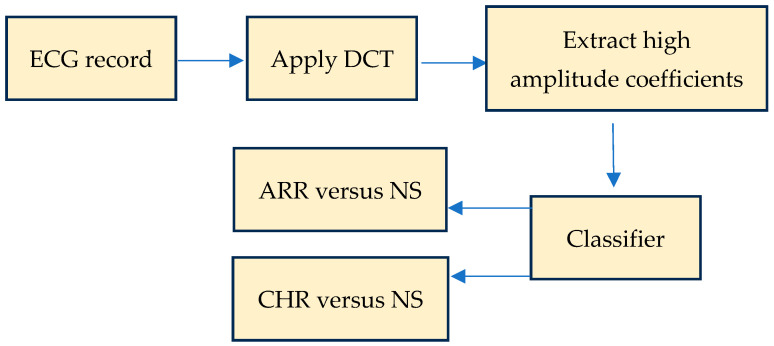
Proposed CAD system to distinguish (*i*) between arrhythmia (ARR) and normal sinus (NS) records, and (*ii*) between congestive heart failure (CHR) and NS records.

For comparison purpose, two other easy to interpret statistical classifiers are investigated, the Naïve Bayes (NB) [13] and the nonlinear support vector machine (SVM) [14]. The NB classifier calculates class membership probabilities based on a conditional independence assumption, and because it requires only a single iteration during the learning process to generate probabilities, it is simple and fast to execute. The SVM employs a nonlinear kernel function to separate data in a high dimensional space. Consequently, it is capable to avoid local minima in the optimization process and offers scalability and generalization capabilities [14]. Figure 1 exhibits the flowchart of our proposed computer-aided diagnosis (CAD) system to distinguish between (*i*) arrhythmia (ARR) and normal sinus (NS) records in one hand, and between (*ii*) congestive heart failure (CHR) and NS records, on the other hand. The classifiers are the kNN algorithm [12,15], Naïve Bayes (NB) [13], and the SVM [14]. 

This study starts by statistically showing that the DCT high amplitude coefficients are significantly different between pathological and normal sinus ECG signals, using the Fisher F-test and two-sample Kolmogorov-Smirnov (KS) test. Second, we show that when the DCT high amplitude coefficients are coupled with a statistical classifier, they are effective in distinguishing between ARR and normal sinus, CHF and normal sinus. Third, the classification results of our three statistical machine learning models are compared in terms of accuracy, sensitivity, specificity and processing time. Finally, the obtained results are compared to recent works [1,2,3,4,5,6,7,10].

### 2.1. The Discrete Cosine Transform

The DCT is a sinusoidal transform with wide application in signal compression and coding. It changes the representation of a signal from the time domain to the frequency domain. More specifically, it describes the signal as a sum of cosine waves with different frequencies and amplitudes. In this regard, it provides a good approximation of the original signal with fewer coefficients [16].

The DCT of a discrete signal x(n) of length N is defined as follows:

(1) $$y\left(k\right)=\sqrt{\frac{2}{N}}\sum_{n=1}^{N}x\left(n\right)\frac{1}{\sqrt{1+\delta_{kl}}}\cos\left(\frac{\pi }{2N}\left(2n-1\right)\left(k-1\right)\right)$$

where *δ_kℓ_* is the Kronecker delta. As mentioned in the Introduction, only the high amplitude coefficients of the transformed ECG signal are retained to form the feature vector that is fed to each statistical classifier since the low amplitude coefficients convey low-pass information with no discrimination relevance regarding ARR and CHR.

### 2.2. The Classifiers

The K-nearest neighbors algorithm (kNN) stores all cases and classifies new ones based on a similarity measure represented by a distance metric. For instance, the goal is to minimize the following function to assign a new object to a particular class:

(2) $$\arg\min\left(d_{e}\left(t,o,k\right)\right)\Rightarrow identify\;\;\;P$$

where *t* is the training data, *o* is the object to be classified, *P* is the assigned class of the new object, *k* is the number of closest neighbors to be considered, and de is a distance. The Euclidean distance was chosen in this study for its effectiveness and reduced computational cost. Parameter *k* is set to one for two reasons. First, it is a conservative value as it makes kNN consider only the closest patterns to a particular class; second, larger values make boundaries between classes less distinct [12,15].

Naïve Bayes (NB) [13] takes a probabilistic approach to determining class membership by modeling the classes of the training data with probability density functions. Then, objects are associated with the most probable class. More precisely, the NB classifier attributes a set of features (*f* = *f*_1_, *f*_2_, …, *f_n_*) to a class c according to:

(3) $$c=\arg\max\left(\mathrm{Prob}\left(c\right)\prod_{i=1}^{n}\mathrm{Prob}\left(f_{i}|c\right)\right)$$

where Prob(*c*) is estimated by the frequency of c in the training data, and Prob(*f_i_*|*c*) is estimated by a Gaussian distribution function. In our experiments, the multivariate multinomial distribution is used to fit the data with the NB method.

The support vector machine (SVM) [14] employs a hyperplane based on the structural risk minimization principle to distinguish between classes. A non-linear kernel function *K* is employed to separate nonlinearly separable data. It is expressed as follows:

(4) $$f\left(x_{i}\right)=sign\left(\sum_{i=1}^{n}y_{i}\alpha_{i}\;K\langle x,x_{i}\rangle +b\right)$$

where *x* is the input, *y* is the output (class label), *α* is the Lagrange multiplier, and *b* is a constant. In our experiments, the SVM with a polynomial kernel is employed as it is less sensitive to outliers, and its order is set to two for fast computation.

## 3. Results

**Figure 2:**
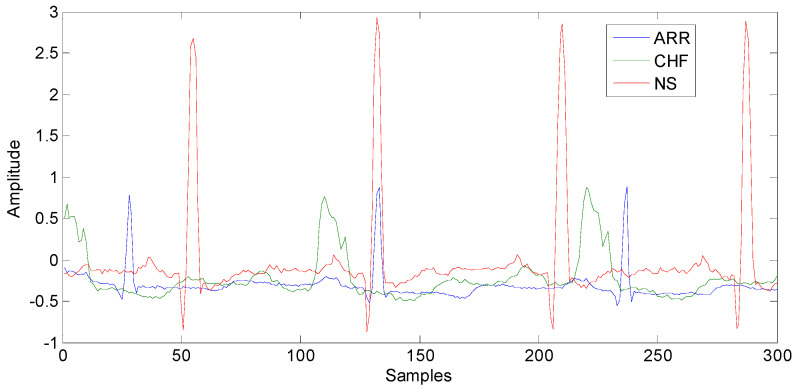
Examples of ECG signals: cardiac arrhythmia (ARR), congestive heart failure (CHF), and normal sinus (NS) rhythm. Only the first 300 samples of each original 65,536-sample ECG are shown.

The classification performance of the proposed approach is evaluated by applying it to ECG records obtained from Physionet [17]. The dataset is composed of 162 ECG records labeled as cardiac arrhythmia (96 signals), congestive heart failure (30 signals) and normal sinus rhythm (36 signals). The signals were resampled at 128 hertz and the length of each one is 65,536 samples. Figure 2 shows examples of the first 300 samples of different original ECG signals. All experiments were performed on Intel(R) Core(TM)2, Duo CPU at 3 GHz in MATLAB© 2024b environment. Machine learning and signal processing toolboxes were used.

**Figure 3:**
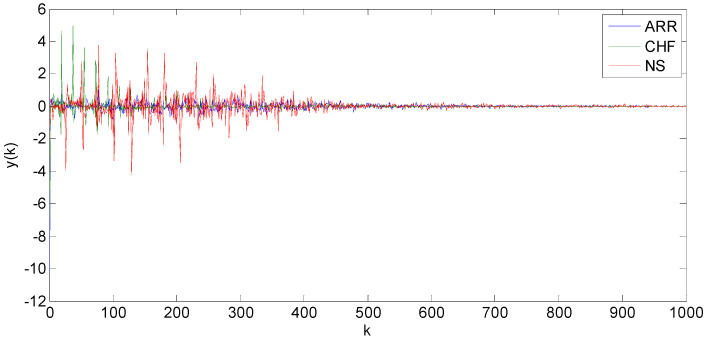
Plots of DCT coefficients y(k) for k = 1 to 1000. ARR, CHF, and NS are respectively cardiac arrhythmia (ARR), congestive heart failure (CHF), and normal sinus rhythm (NS).

The DCT was applied to each ECG signal with the number of coefficients arbitrarily set to 1000 in Equation (1). As a result, each original 65,536-sample ECG signal was compressed to 1000 DCT coefficients, of which only the first half (the high amplitude coefficients) were used to train the three statistical classifiers. Figure 3 provides example plots of the DCT coefficients from pathological and normal ECG signals, confirming that most of the information content lies in the first 500 values.

In a preliminary experiment, for each type of ECG signal, we averaged the vectors of the DCT-based high amplitude coefficients to obtain three vectors of 500 elements. Then, we performed two statistical tests to check the differences between average ARR and average NS rhythm and between average CHF and average NS rhythm. The two-sample F-test for equal variancewasutilized in our experiments at a 5% statistical significance level to test whether or not high amplitude coefficients in abnormal and normal ECG signals have similar variability. The resulting F-statistics and probability values(*p*-values) are provided in Table 1. According to the obtained *p*-values, the variability for the high amplitude coefficients is different across ARR and NS rhythm since the computed *p*-value is less than a 5% significance level. Similarly, the variability of high amplitude coefficients across CHF and NS rhythm is also different.

In addition, the two-sample Kolmogorov–Smirnov (KS) test is performed to test the null hypothesis that the high amplitude coefficients in the abnormal and normal ECG signals have similar distributions. The KS-statistic and *p*-value of the KS-test are provided in Table 1. The obtained *p*-values show that the distribution of the ARR high amplitude coefficients is different from that of NS rhythm high amplitude coefficients at the 5% statistical significance level. Similarly, the null hypothesis of similar distribution of high amplitude coefficients across CHF and NS rhythm is also rejected.

The results from the F-test and KS-test show strong evidence that high amplitude coefficients are different across pathological and normal ECG signals. Therefore, these representative ECG characteristics could be considered as feature candidates for the ensuing classification task. In this regard, Table 2 compares the obtained classification results by kNN, NB, and the nonlinear SVM after training to discriminate between ARR and NS rhythm and to discriminate between CHF and NS rhythm. The classification experiments were conducted using 10-fold cross validation. Accordingly, the average and standard deviation of accuracy (correct classification rate), sensitivity (true positive rate), and specificity (true negative rate) wereused to assess the performance of each statistical classifier. Also, the processing time of the 10-fold cross validation method was used to evaluate the complexity of each classifier in terms of computational time.

As shown in Table 2, for both classification problems (ARR versus NS rhythm and CHF versus NS rhythm), kNN outperforms both NB and the SVM in terms of accuracy and specificity. Also, for both classification problems, in terms of sensitivity, kNN performs better than NB, but the SVM achieves perfect sensitivity. Finally, kNN is the fastest, followed by NB and the SVM.

## 4. Discussion

Electrocardiography is a popular noninvasive technique for monitoring human heart electrical activity with various biomedical engineering applications, including ECG complexity analysis [18,19,20,21,22,23,24,25,26], pathology detection [1,2,3,4,5,6,7,8,9,10,27] and signal denoising [28]. In this study, three statistical classifiers are employed to classify normal and pathological ECG signals when the DCT is applied to extract a features vector consisting of the lower half of the obtained coefficients. Those high amplitude coefficients are used to characterize the original ECG signal patterns and kNN, NB, and nonlinear SVM classifiers are trained with them for ECG signal classification following two different scenarios: cardiac arrhythmia versus normal sinus rhythm, and congestive heart failure against normal sinus rhythm. 

Using the DCT has several advantages in addition to fast response and signal compression. For instance, contrary to the wavelet transform, there is no need to select a pre-determined wavelet function for the analysis of the original signal, and no need to find the optimal decomposition level. In the DCT framework, only the number of coefficients to be extracted must be specified. Since we are concerned with a fast and reliable model for ECG signal analysis and classification without a particular pre-processing step of the underlying signal, we focused on coupling the DCT with a fast-learning statistical classifier. The kNN classifier was chosen for the purpose, given its better overall performance in comparison to the NB and SVM alternatives; both chosen as secondary classifiers. As statistical classifiers, the main advantages of kNN, NB, and SVM are fast computation and interpretability, in contradistinction to approaches such as artificial neural networks. Moreover, evolutionary/heuristic techniques are not considered in the current work as they require design of appropriate architecture, fine tuning of several parameters, and are difficult to interpret. 

To assess the performance of each statistical classifier, we chose10-fold cross-validation, which is a well-known method for evaluation to reduce the bias related to the random sampling of the training and test sets. In this respect, in 10-fold cross validation, the whole dataset is randomly split into 10 distinct subsets (folds) of approximately equivalent size. Each statistical classifier is trained and tested 10 times. Specifically, each one is trained on all but one of the folds and tested on the remaining single fold.

Our experiments were applied to a dataset from Physionet [17] containing ECG signals labeled cardiac arrhythmic, congestive heart failure, and normal sinus rhythm. To the best of our knowledge, our study is the first to use this new dataset. Therefore, our work constitutes the basis for future studies and comparisons whenever this dataset is used.

It is worth noting that applying the DCT to the entire dataset takes 0.3889 s (on average 0.0024 s for a single ECG signal) and that all three statistical classifiers learned and classified the whole dataset following the 10-fold cross-validation protocol with exceptional speed, for instance, less than a second to five seconds as indicated in Table 2. In short, signal compressing by the DCT and classification are not computationally intensive, which makes the approach attractive for real biomedical applications.

For a general evaluation of our approach, a comparison in terms of accuracy between the results obtained herein and those reported in other recent studies is provided in Table 3. When compared to recent works on the classification of ARR versus NS rhythm, our approach yielded comparable accuracy with the limited computational complexity of [O(n × log(n))] since the DCT is computed with the fast Fourier transform algorithm. When compared to recent works on the classification of CHR versus NS rhythm, our approach achieved better accuracy as indicated in Table 3. More importantly, DCT-HAC-kNN and DCT-HAC-SVM yielded perfect sensitivity in both classification problems. This is particularly remarkable knowing that physicians are more concerned with the detection of true positives with very high accuracy for early and appropriate treatment. For instance, the authors in [6] reported 98.6% sensitivity when distinguishing between CHR and NS rhythm, while the authors in [8] reported 87% sensitivity for the same classification problem. Our approach appears to be more appropriate to detect ARR and CHF, in this respect. In addition, it is based only on DCT for both signal compression and features extraction, while previous works [1,2,3,4,5,6,7,8,9,10] require various signal transformations [3,4,5,10], features from different spaces [1,3,4,5,7,10], and usage of two different dimensionality reduction techniques [3]. As shown in Table 3, there are various features extraction methods applied to ECG records, including morphological features [1], coefficients of differential equations [2], statistical features obtained from spectral analysis [3], random projection matrix [4], higher-order statistics and sample entropy estimated from the domain of improved complete ensemble empirical mode decomposition [5], static indices [6], time domain variability [7], and time domain statistics [10]. The main advantage of our approach is to be on DCT for ECG analysis and features extraction. Indeed, DCT has several advantages including an excellent capability to compact the energy of signal into a few coefficients while preserving very good representation of the signal, being real valued transform which means no need to introduce any complex numbers or phase information, and being based on a fast algorithm; hence, the DCT algorithm is computationally efficient. In sum, the DCT is easy to implement, fast and effective in revealing high-frequency oscillations in ECG used to discriminate statistically and significantly between unhealthy and healthy subjects as shown in Table 1. Furthermore, the classifiers considered in our study achieved high performance when trained with DCT high frequency components as shown in Table 2. 

Another advantage of our approach is that the classification of each statistical classifier can be interpreted. For instance, the good performance of kNN over NB and the SVM can be explained by the importance of capturing the distance between patterns and labels in classification tasks and because of its effectiveness in the automatic classification of complex biomarkers [29,30]. The good performance of NB over the SVM in classifying CHF versus NS rhythm suggests that the probability density function in assigning class membership probabilities of patterns is more significant than structural risk minimization indistinguishing between CHR and NS rhythm. In contrast, the good performance of the SVM over NB in classifying ARR versus NS rhythm suggests that structural risk minimization indistinguishing between ARR and NS rhythm is more effective than estimating probability density functions. It is worth noting that deep learning models like the Manifold regularization-based deep convolutional autoencoder [31] would be useful for distinguishing between ECG records as it combines Manifold regularization, convolutional layers, and autoencoders for better deep feature extraction and classification.

## 5. Conclusions

This work presented a simple model for the detection of abnormal rhythms in ECG signals based on kNN and high amplitude coefficients obtained by the DCT. The cardiac arrhythmia and congestive heart failure detection model not only offers excellent classification performance compared to the NB and SVM methods trained with the same coefficients but also demonstrated superior detection performance when compared with some other works.

For future work, three issues can be investigated. First, the effects of the number of high amplitude coefficients on accuracy will be examined for a full understanding of the mechanics of pathological ECG signals. Second, another interesting route to be explored is to employ a feature selection scheme to select the most valuable DCT-based high amplitude coefficients in an attempt to improve overall accuracy. Third, as we did not evaluate our approach by classifying SN, ARR, and CHF simultaneously, this appealing issue will be left for future study.

## Figures and Tables

**Table 1:** Results from statistical tests.

	*F*-Statistic	*p*-Value
**ARR versus NS**	9.4150	0.0000
**CHR versus NS**	10.5205	0.0000
	**KS-Statistic**	***p*-Value**
**ARR versus NS**	0.1380	0.0000
**CHR versus NS**	0.1080	0.0053

**Table 2:** Comparison of performance of classifiers.

Classifiers	Acc. (%)	Sens. (%)	Spec. (%)	Time (s)
**ARR versus NS**				
*k*NN	96.61 ± 0.0368	99.00 ± 0.0131	90.22 ± 0.1048	0.6321
NB	92.39 ± 0.0182	94.64 ± 0.0270	86.17 ± 0.0788	0.7318
SVM	94.61 ± 0.0477	100	80.08 ± 0.1754	4.8862
**CHR versus NS**				
*k*NN	99.39 ± 0.0192	99.67 ± 0.0105	99.17 ± 0.0264	0.2684
NB	95.68 ± 0.0364	98.55 ± 0.0246	93.31 ± 0.0518	0.6181
SVM	87.54 ± 0.0729	100	76.74 ± 0.1335	0.8443

**Table 3:** Comparison with other studies.

Studies	Features	Classifier	Accuracy
**ARR versus NS**			
[1]	Morphological features + RR timing	Modular neural network	96.02%
[2]	Coefficients of differential equations	Genetic fuzzy system	93.34%
[3]	Spectral analysis + statistical features + PCA + Fisher score	SVM	98.60%
[4]	Random projection matrix + weighted RR timing	SVM	98.46%
[5]	ICEEMD + HOS + SE	AdaBoost	98.6%
Current work	High amplitude coefficients of DCT	*k*NN	96.61% ± 0.0368
**CHR versus NS**			
[6]	Static indices	SVM	98.31%
[7]	Time domain variability statistics + RR statistics	Ensemble bagged trees	99.5%
[10]	Time domain statistics + frequency domain statistics + nonlinear statistics	SVM	90.95%
Current work	High amplitude coefficients of DCT	*k*NN	99.39% ± 0.0192

## Data Availability

The data were obtained from Physionet [17]. Accessed on 1 November 2024.
